# An overview of structural equation modeling: its beginnings, historical development, usefulness and controversies in the social sciences

**DOI:** 10.1007/s11135-017-0469-8

**Published:** 2017-01-09

**Authors:** Piotr Tarka

**Affiliations:** Department of Market Research, Poznan University of Economics, al. Niepodleglosci 10, 61-875 Poznan, Poland

**Keywords:** Structural equation modeling, Development and usefulness of SEM, Controversies of SEM, Areas of applications in social science research

## Abstract

This paper is a tribute to researchers who have significantly contributed to improving and advancing structural equation modeling (SEM). It is, therefore, a brief overview of SEM and presents its beginnings, historical development, its usefulness in the social sciences and the statistical and philosophical (theoretical) controversies which have often appeared in the literature pertaining to SEM. Having described the essence of SEM in the context of causal analysis, the author discusses the years of the development of structural modeling as the consequence of many researchers’ systematically growing needs (in particular in the social sciences) who strove to effectively understand the structure and interactions of latent phenomena. The early beginnings of SEM models were related to the work of Spearman and Wright, and to that of other prominent researchers who contributed to SEM development. The importance and predominance of theoretical assumptions over technical issues for the successful construction of SEM models are also described. Then, controversies regarding the use of SEM in the social sciences are presented. Finally, the opportunities and threats of this type of analytical strategy as well as selected areas of SEM applications in the social sciences are discussed.

## Introduction

One of the main goals of research in the social sciences, i.e., in the context of recognizing particular concepts and events, is to explain and to predict, in a valid manner, the specific behavior of an individual, group of people or organization. Researchers, by recognizing a number of conditions in which the individual, society or organization exists, can, within certain limits, identify particular development trends and describe the details concerning their existential sphere. As a result, researchers can define and discover the vital factors and relationships which set trends in a given society. However, the goal of the social sciences is not only to conduct an elementary statistical description and to recognize individual factors and behaviors (which are involved in a specific social situation), but also to determine the cause-effect linkages among the scientific areas (i.e., variables) of interest. Because of the complexity of social reality, i.e., the latent character of many social phenomena, sophisticated methods and techniques of statistical data analysis are required, both of which refer to causal analysis and the procedures of encompassing many variables based on *Structural Equation Modeling*—SEM. In the statistical sense, this model refers to a set of equations with accompanying assumptions of the analyzed system, in which the parameters are determined on the basis of statistical observation. Thus, structural equations refer to equations using parameters in the analysis of the observable or latent variables (Jöreskog and Sörbom 1993). In the latter case of variables, their examples could be such theoretical constructs as: intelligence, alienation, discrimination, socialization, motives of human behavior, personal fulfillment, aggression, frustrations, conservatism, anomie, satisfaction, or attitudes. In the economic sense, these can also be: prosperity of a geographic region, social-economic status, satisfaction from purchased products, approval of products, and improvement of economic conditions. All in all, the measurement of such latent constructs is conducted indirectly, mostly with the use of a set of observable variables and via observation of the causal effects in SEM between respective latent variables.

## Spearman’s factor analysis as a primary source of structural equation modeling development

The dissemination and development of structural modeling (SEM) was the consequence of the growing needs of both academic researchers and social science practitioners who were looking for effective methods in order to understand the structure and interactions of latent phenomena. For years, human motivations have been the source of development for many analytical procedures, thus the early beginnings of SEM development should be reconstructed indirectly on the basis of Spearman’s works (1904, 1927), as he laid the foundations for SEM by constructing the first factor model which later became an important measurement part of the more general SEM analytical strategy. Spearman (1904) is often cited in the literature as the founding father of factor analysis, even though one year earlier Pearson (1901a) published a paper on fitting planes by orthogonal least squares, which was the foundation for principal component analysis that was also applied to the analysis of correlation matrices by Hotelling (1933). What Spearman did exactly was to measure general cognitive abilities in humans by using models of the so-called factor analysis. In his work he claimed that observable statistical relationships among disparate cognitive test scores can reflect latent levels of human intelligence that are common for all tests and specific intelligence factors related to each test score. Then he specified a two-factor theory of intelligence in which all mental processes involved a general factor and a specific factor. Therefore, Spearman’s work (1904) marked the beginning of the development of factor models which later became the key for the construction of measurement models used in SEM. Although in his research Spearman focused on the ‘factor model’, his pioneering works gave meaning to and revolutionized the thinking of many researchers about the measurement of latent variables which, in light of the True Score Theory (see Gulliksen 1950), can today be viewed as a peculiar constraint in the context of the measurement due to random and nonrandom errors.

Thurstone (1935) criticized Spearman’s work because it was mainly focused on the two-factor theory. Thurstone noted that a vanishing tetrad difference implies a vanishing second-order determinant of the matrix of observable variables, and therefore decided to extend it to the vanishing of higher-order determinants as a condition for more than one factor. Later he generalized the result as the number of common factors that was determined by the rank of the matrix of observables (Harman 1960). Next, Thurstone (1935) developed the centroid method of factoring a correlation matrix (as a pragmatic compromise to the computationally-burdensome principle axis method). Moreover, he developed a definition of a simple structure for factor analysis based on five principles (the most important of which was to minimize negative loadings and maximize zero loadings) to facilitate interpretation and to insure that the loadings were invariant to the inclusion of other items. From that moment on all scholars’ main interest in the domain of factor analysis were directed at various methods of rotation, such as Kaiser’s (1958) Varimax orthogonal rotation. Thurstone also contributed to the idea of rotation, which was based on the oblique solution allowing factors to be correlated, but in reality it was credited to Jennrich and Sampson (1966), who developed a computational method of achieving an oblique rotation. Jennrich, while collaborating with Clarkson (1980), also diagnosed standard errors of the rotated loadings. In the end, the problem with factor rotation was solved when confirmatory factor analysis was invented, in which the number of common factors (latent variables) and the pattern of loadings (including constraints set on the loadings) could be specified in advance.

To sum up, Spearman’s works did not refer to the statistical assumptions of hypothesis testing in terms of determining the structure of a factor model but to intuitive theoretical assumptions of the investigated phenomenon. Other works, which in later years contributed to the development of factor analysis, generally concerned such issues as the multiple factor model (Thurstone 1935, 1938, 1947), the scope of knowledge used by the researcher before the rotation of factors (Mosier 1939), and statistically optimal methods of factor extraction (Lawley 1940), constraints imposed on the factor models, e.g., by setting the factor loadings to zero (Anderson and Rubin 1956; Lawley 1958). Finally, thanks to the work of Tucker (1966), the differentiation between exploratory and confirmatory factor analysis appeared for the first time in the literature. Also, at that time the first studies on the structure of covariance (Wilks 1946; Votaw 1948) were conducted.

## Wright’s path analysis and early years of SEM growth as an analytical strategy

Real works concerning the idea of Structural Equation Modeling were actually initiated by Wright (1918, 1921, 1934, 1960a, b),^1^ a geneticist who used an approach based on path analysis with the structural coefficients estimated on the basis of the correlation of observable variables, although he also worked with latent variables. What truly made Wright develop path analysis was the fact that he was dissatisfied with the results of the partial correlation analysis that was being conducted which remained far from a causal explanation. Consequently, he developed path analysis to impose a causal structure, with structural coefficients, on the observed correlations. However, Wright was not only an originator of path analysis as the analytical strategy but also the originator of either a graphic or diagrammatic representation of relations between variables included in this type of analysis. By constructing path diagrams he was able to quickly decompose the correlations into various causal sources, such as direct effects, indirect effects, common causes, and the like. Thus thanks to his research it was possible to identify total, direct and indirect causal effects, although initially in Wright’s models the causal flow was assessed from the perspective of only one direction, which means that the models of that time had a recursive character. To sum up, his main contribution was to present, in a path diagram, how correlations between variables can be linked with model parameters. Then he showed how the equations of the model can be used to estimate direct, indirect, and total effects of one variable influencing the other variable.

In 1960 Wright (1960a, b) expanded the methods of finding model correlations, which marked the beginning of non-recursive models, that had previously been developed in the field of econometrics (Frish and Waugh 1933; Frish 1934; Haavelmo 1943). The non-recursive models assumed the simultaneous influence of a few variables on other variables with possible feedback loops as well as disturbance covariances (see the works of Klein 1950; Goldberger 1964; Theil 1971). Finally, among Wright’s models there appeared a model of multiple causal indicators (later known as the MIMIC model). Wright’s estimation method was essentially a method of moments which followed the intuitive principle of estimating a population moment by using the sample analog moment. Although Wright lacked a principle for reconciling multiple ways of expressing a path coefficient in terms of sample moments in overidentified models, he did check to see if they were close and acknowledged the potential gains in efficiency and reduced standard errors from using full information (Matsueda 2012). Interestingly, Wright’s work was long ignored by some statisticians, as he was criticized for introducing the differentiation between causes and correlations. This criticism came mainly from statisticians centered around Pearson and Fisher’s school of statistics.

Econometrics, on the basis of Wright’s work, introduced a rigorous condition of meeting the requirements concerning the correct formulation and estimation of SEM models (Li 1975). This issue was particularly focused on problems of model identification (Koopmans 1949; Wald 1950) and on alternative methods of SEM parameter estimation (Goldberger 1972). The SEM approach in econometrics was mainly promoted by Haavelmo (1943), Koopmans (1945), and Frisch and Waugh (1933). These scholars made a milestone in providing an understanding of the principles of SEM by defining the ‘structural relation’ as ‘the theoretical relation postulated a priori’ in a single-equation multivariate linear model in which the partial regression coefficient represented a ‘structural coefficient’. Frisch (1934) was, however, sceptic of the use of probability models for economic data, which were rarely the result of a sampling process, and of OLS (Ordinary Least Squares) regression, because measurement errors existed not only in the dependent variables but also in the independent variables. Frisch treated observable variables as fallible indicators of the latent variables to distinguish ‘true relations’ from ‘confluent relations’. Haavelmo (1943, 1944), on the other hand, contributed to the development of SEM by specifying a probability model for econometric models and concisely described the Neyman–Pearson (1933) approach to hypothesis testing by using the probability approach for estimation, testing, and forecasting. He also distinguished between two models of the source of stochastic components, i.e., errors-in-variables models, as emphasized by Frisch (1934), and random shock models, as introduced by Slutsky (1937). This framework is often defined as the “probabilistic revolution” in econometrics and has had a lasting impact on the field, particularly in cementing the Neyman-Pearson approach to inference over others, such as Bayesian approaches. Finally, Haavelmo (1943, 1944) advanced SEM by proving that OLS estimates are biased in a two-equation supply–demand model and distinguished between the structure for equations and what Mann and Wald (1943) termed as the reduced-form equation. He applied the maximum likelihood (ML) estimation to the system of equations, showing its equivalence to OLS when applied to the reduced form, and further specified the necessary conditions for identification in terms of partial derivatives of the likelihood function (Matsueda 2012). Later, Koopmans et al. (1950), who also worked in the ‘Cowles Commission’,^2^ helped to solve major problems of identification, estimation, and testing of SEM models.

In another field of science, namely in sociology, Blalock (1961a, b, 1963, 1964, 1971), taking inspiration from the works of biometricians and econometricians, made the first attempts to combine the simplicity of presentation which path analysis offered with the rules of defining equation systems that were used in econometrics. In the sociological literature, however, the main credits was ascribed to Duncan (1966), who worked on the problems of correlations and their applications in path analysis to recursive models based on class values, occupational prestige, and synthetic cohorts.^3^ Later, in 1975, Duncan authored an excellent text for path analysis and structural equation models in which he echoed Frisch and Haavalmo’s concept of autonomy—“the structural form is that of parameterization, in which the coefficients are relatively unmixed, invariant, and autonomous” (Duncan 1975; p. 151). He also distinguished between forms of social change from trivial changes in sampling or exogenous variables (which leave the structural coefficients intact), to deeper changes in the structural coefficients (which provide an understanding for the explanation of SEM models) and changes in the model’s structure itself, and provided important hints for applying the structural models. As he claimed (Duncan 1975; p. 150), “researchers should not undertake the study of structural equation models in the hope of acquiring a technique that can be applied technically to a set of numerical data with the expectation that the result will automatically be researched”.

Blalock (1969) concentrated in his work mainly on multiple-indicator causal models, in particular attempting to find tetrad-difference restrictions on observed correlations that provide a way of testing the models. Blalock (1961a, b; p. 191) also stressed that “path analysis can be boiled down to sciences in which there are no strict rules of using experiments”, although this statement will be questioned later in the literature (see the next sections). Finally, Blalock, while working on the causal models, elaborated Simon’s (1954) approach of making causal inferences from correlational data. The latter author (Simon 1954; p. 41) argued that “determination of whether a partial correlation is or is not spurious can only be reached if a priori assumptions are made that certain other causal relations do not hold among the variables”. Simon (1954) described these conditions in all possible three-variable models, which were extended by Blalock (1961b, 1962) to a four-variable model.

Finally, in psychology, SEM as an analytical strategy was introduced successively, mainly thanks to the works of Werts and Linn (1969), and Issac (1970). However, their works did not cause any breakthrough interest of psychologists at that time around the SEM strategy because the assumptions of SEM models were technically complex and few researchers were able to understand them. Psychology, and more specifically psychometrics, marked the beginning of SEM models, but indirectly by laying the theoretical grounds for the Classical Test Theory (CTT) and measurement models. In fact, psychology developed a more theoretical background for factor analysis.

## Influence of computer software on structural equation modeling

Although considerable growth of interest in SEM models was caused largely thanks to the works of Goldberger (1971, 1972) and to the publication titled *Structural Equation Models in Social Sciences* (Goldberger and Duncan 1973), which was the effect of an interdisciplinary conference organized in 1970 featuring economists, sociologists, psychologists, statisticians, and political scientists (from the Social Science Research Council) that was devoted to issues of structural equation models, in practice the true development of structural models results from the dynamic development of statistical software and synthetic combination of measurement models with structural models, which was expanded in the field of psychometrics and econometrics. Interestingly, although the methodological concepts related to SEM which appeared in the works of Jöreskog (1970, 1973), Keesling (1972) and Wiley (1973) were independently proposed (i.e., the studies were simultaneously conducted by the three researchers), in the literature mainly Jöreskog (1973) has been credited with the development of the first SEM model (including computer software (LISREL).

The LISREL was the first computer project; however, Jöreskog along with two other authors (Gruavaeus and van Thillo) had previously invented the ACOVS, which was a general computer program for the analysis of covariance structures (Jöreskog et al. 1970). Thus the ACOVS was virtually a precursor of the LISREL (Jöreskog and Sörbom 1978). Moreover, work on the LISREL actually began in 1972, when Jöreskog left the Educational Testing Service at Princeton University to take over a professorship position in statistics at the University of Uppsala in Sweden. His academic colleague, Sörbom, prepared all of the programming schemas in the LISREL and developed the method to estimate latent means. However, before he began his work Jöreskog profited from Hauser and Goldberger’s book chapter (1971) on the examination of unobservable variables which was, at that time, an exemplar of cross-disciplinary integration and which drew on path analysis and moment estimators (from Wright and other sociologists), factor models (from psychometrics), and efficient estimation and Neyman-Pearson hypothesis testing (from statistics and econometrics). Hauser and Goldberger focused on the theory of limited information estimation by trying to disclose the real facts behind the model system of structural equations as estimated by maximum likelihood. Jöreskog (1973) summarized their approach, presented the maximum likelihood framework for estimating SEM, and developed the above-mentioned computer software for empirical applications. Furthermore, he showed how the general model could be applied to a myriad of important substantive models.

A general advantage of the model proposed by Jöreskog was the explicit possibility of practical application, as the general model at that time contained all linear models that had been specified so far. In other words, the model’s usefulness was in its generality and in the possibilities it offered in practical applications. The first sub-model resembled the econometric configuration of simultaneous equations but was designed for latent variables, whereas the second sub-model was a measurement model which included latent variable indices just as in the psychometric theory of factor analysis. Simultaneously, apart from its being universal, the structural model was expressed in the form of matrices containing model parameters. Thus the model could be successfully applied in many individual research problems (Jöreskog 1973). Finally, Jöreskog (1971) also generalized his model and virtually all of his academic papers to estimate the model in multiple populations showed how the LISREL could be applied to simultaneous equations, MIMIC models, confirmatory factor models, panel data, simplex models, growth models, variance and covariance components, and factorial designs. In the following years this model evolved into further alternative solutions, such as COSAN—*Covariance Structure Analysis* (McDonald 1978), LINEQS—*Linear Equation Model* (Bentler and Weeks 1980), RAM—*Reticular Action Model* (McArdle and McDonald 1984), *EzPath* (Steiger 1989), or RAMONA (now a part of SYSTAT software—see the work of Browne and Mels 1990).

Besides the LISREL, the real boom in SEM software development came along with many other commercial computer packages, such as EQS (Bentler 1985), LISCOMP, which was renamed MPLUS (Muthén 1987a; Muthén and Muthén 1998), AMOS (Arbuckle and Wothke 1999), PROC CALIS (in SAS), HLM (Bryk et al. 1996), SIMPLIS (Jöreskog and Sorböm 1996), and GLAMM (Rabe-Hesketh et al. 2004; Skrondal and Rabe-Hesketh 2004), as well as freeware packages related to an R (open source) statistical environment, such as OpenMX (Development Team 2011), SEM package (Fox 2006), or LAVAAN (Rosseel 2012). The common advantage of all of this software is that it offers highly advanced and fast-speed computational solutions, e.g., in conducting the simulation of experimental plans, and allows to more precisely confirm the correlations between the analyzed variables, together with the available possibility of testing cause-effect relationships, e.g. Bentler’s EQS software (1985) can be applied on the basis of syntax. In contrast, in AMOS the path diagram’s flexible graphical interface can be used instead of syntax. OpenMx, which runs as a package under R and consists of a library of functions and optimizers, supports the rapid and flexible implementation and estimation of SEM models. In consequence, it allows for estimation of models based on either raw data (with FIML modeling) or on correlation or covariance matrices. OpenMx can also handle mixtures of continuous and ordinal data (Neale et al. 2016). Likewise as OpenMX, the SEM package provides basic structural equation modeling facilities in R and includes the ability to fit structural equations into observed variable models via the two-stage least squares procedure and to fit latent variable models via full information maximum likelihood assuming multivariate normality. Finally, with the LAAVAN package a large variety of multivariate statistical models can be estimated, including path analysis, confirmatory factor analysis, structural equation modeling and latent growth curve models.

Many of the above advancements in software took place at a time when the automated computing process offered substantial upgrades over the existing calculator and analog computing methods that were available then. Admittedly, advances in computer technology have enabled many professionals, as well as novices, to apply structural equation methods to very intensive analyses based on large datasets which refer to often complex, unstructured problems (see the discussion in the work of Westland 2015).

## Progress in SEM model parameter estimation methods

While the early 1970s were characterized by achievements in generalizing and synthesizing models developed in econometrics, sociometrics, psychometrics and biometrics, the late 1970s and 1980s made a huge advancement in parameter estimation methods. However, we must remember that early applications of path analysis were based only on ad hoc methods in the estimation of model parameters. The formal approach to the estimation of an SEM model is owed to the work of Goldberger (1964), who developed the characteristics of estimators for models with observable variables, and to statisticians such as Anderson and Rubin (1956), Lawley (1967) as well as Jöreskog (1969, 1970, 1973). Also Bock and Bergman (1966) developed covariance structure analysis in estimating the elements of covariance of observable variables having multidimensional, normal distribution and a latent character.

Anderson and Rubin (1956) created a limited information maximum likelihood estimator for parameters of a single structural equation which indirectly included a two-stage least squares estimator and its asymptotic distribution.^4^ However, as Browne argued (Browne 2000b, p. 663), “the computational procedures were not available until the nested algorithms involving eigenvalues and eigenvectors and imposing inequality constraints on unique variance estimates were discovered independently by Jöreskog (1969) and by Jennrich and Robinson (1969)”. Jöreskog (1973), in his breakthrough article, proposed use of the maximum likelihood estimator but, as he himself admitted, a numerical procedure for obtaining the ML estimates under certain special cases had first been delivered by Howe (1955) and Lawley (1940, 1943, 1958). This procedure was also related to confirmatory factor models (Lawley and Maxwell 1963).

The ML estimator was often a subject of criticism in the literature because of the unrealistic assumptions of the continuous observable, the latent variables (e.g., multivariate normal distributions), and the large sample sizes which were needed to meet the asymptotic properties of this estimator and efficient testing. In the last case, although large sample sizes may generally provide sufficient statistical power (see e.g., Kaplan 1995) and precise estimates in SEM, there is no clear consensus among scholars as to the appropriate methods determining adequate sample size in SEM. In the literature, only selective guidelines have appeared (on the basis of conducted simulation studies, e.g., Bentler and Chou 1987; collected professional experience, MacCallum et al. 1996; or developed mathematical formulas, Westland 2010) to determine appropriate sample size. Most of them refer to problems associated with the number of observations falling per parameter, the number of observations required for fit indices to perform adequately, and the number of observations per degree of freedom.

Another issue is the effect of categorization of observable variables (e.g., on a Likert scale) which one can often encounter in social studies. Boomsma (1983) argued that although the SEM models, i.e., their estimators, behave more or less properly for samples exceeding 200 observations, the skewness of the categorized variables may cause problems, such as spurious measurement error correlations and biased standardized coefficients. This ‘abnormality’ helped to promote two scholars, Browne and Muthén, among the academic society. The former proposed a generalized least squares estimator (GLS) which allowed to release some of the ML’s strict assumptions (Browne 1974), however, it was the further work of Browne (1982, 1984) that turned out to be particularly vital. Browne contributed to SEM mainly by developing an asymptotic distribution-free estimator ADF (otherwise known as WLS—Weighted Least Estimator) in which he presented the asymptotic covariance matrix and asymptotic Chi-square test statistic as well as an estimator for elliptical distributions which had zero skewness but the kurtosis departed from multivariate normality. Browne’s ADF estimator (1984) was further included in Bentler EQS software (1985) and other software, and examined on the basis of finite sample properties, e.g., the findings indicated that the ADF estimator behaved best in very large samples (1000, 2000), which in the end turned out to be a disadvantage of the estimator itself, as researchers rarely conduct studies that include samples of that size in the social sciences (e.g., in survey research).

The works of Browne (1984) became a crucial element in the development of models with ordinal, limited, and discrete variables, whose original creator was Muthén (1983, 1984). The success of Muthén’s approach lay in the estimation of scale-appropriate correlation coefficients (e.g., polychoric and polyserial) and then in the application of Browne’s ADF estimator (1984).^5^ Then researchers could bind, for example, the ordinal variable with a normally-distributed continuous latent variable through the so-called threshold model. Simultaneously, work was continued on factor models for dichotomous variables, e.g., Bock and Lieberman (1970) used tetrachoric correlations and an ML estimator for a single factor model, and Christofferson (1975) generalized this to multiple factors using a GLS estimator (see also Muthén 1978). Muthén (1979) subsequently developed a multiple-indicator structural probit model, while Winship and Mare (1983) showed how to apply multivariate probit models (estimated by ML) to multiple-indicator structural equation models and path analysis.

In the past decade a large number of simulations appeared allowing to identify the characteristics of the distribution of variables which may influence the empirical behavior of estimators in relatively small research samples (Boomsma and Hoogland 2001). Particularly work on overcoming the lack of normality in variable distribution (including Muthén’s earlier papers from the years 1983, 1984)^6^ went in two directions: one direction has allowed to construct robust estimators based on scaled Chi-square statistic and robust standard errors in using ML estimation (Hu et al. 1992; Satorra and Bentler 1988, 1994, 2001; Curran et al. 1996; Yuan and Bentler 1998), while the second direction has used the strategy of bootstrap resampling to correct standard errors (for a review of this methodology, see Yung and Bentler 1986; Bollen and Stine 1992; Nevitt and Hancock 2001). The simulation work that was conducted so far (e.g., Fouladi 1998; Hancock and Nevitt 1999; Nevitt and Hancock 2001) suggested that in terms of bias a standard ‘naïve’ bootstrap mechanism works at least as well as robust adjustments to standard errors. However, Nevitt and Hancock (2001) suggested that standard errors may be erratic for a sample size of 200 or fewer, hence samples of 500–1000 may be necessary to overcome this problem. The complexity of the SEM model should also be diagnosed, because the Nevitt and Hancock (2001) simulations were based only on a moderately complex factor model (i.e., smaller sample sizes may be acceptable for simpler models). Finally, an alternative bootstrapping approach was introduced into the literature by Bollen and Stine (1992) for estimation of the Chi-square which seems to adequately control the type I error, though with some some cost to statistical power (see Nevitt and Hancock 2001).

## Contemporary advancements in structural equation modeling

The transformations that SEM has experienced in recent years have caused further generalizations of this analytical strategy. Thanks to the works of Bartholomew (1987), Muthén (1994, 2001, 2002) and Skrondal and Rabe-Hesketh (2004), SEM has become a very general latent variable model which, together with the linear mixed model/hierarchical linear model, is the most widely recognized statistical solution in the social sciences (see the works of Muthén and Satorra 1995; MacCallum and Austin 2000; Stapleton 2006). Most of these contemporary advancements were made in the area of latent growth curve and latent class growth models for longitudinal data, the Bayesian method, multi-level SEM models, meta-SEM-analyses, multi-group SEM models, or algorithms adopted from artificial intelligence in order to discover the causal structure within the SEM framework. Below we will discuss some of these contemporary developments.

### The Bayesian method in SEM

The Bayesian method created a different perspective for structural equation modeling, in particular in the context of the estimation procedures. From the Bayesian point of view, the estimation process is less demanding in the context of deducing the values of population parameters and is more about updating, sharpening, and refining beliefs about the empirical world. Thus with the Bayesian approach we use our ‘background knowledge’ (encompassed in what is called ‘a priori’) to aid in the model’s estimation.

Bayesian analysis brought many benefits to SEM. One of them is the opportunity to learn from the data and to incorporate new knowledge into future investigations. Scholars need not necessarily rely on the notion of repeating an event (or experiment) infinitely as in the conventional (i.e., frequentist) framework; instead, they can combine prior knowledge with personal judgment in order to aid the estimation of parameters. The key difference between Bayesian statistics and conventional (e.g., ML estimator) statistics is the nature of the unknown parameters in the statistical model (Van de Schoot and Depaoli 2014). Also, the Bayesian method helped to improve the estimation of complex models, including those with random effect factor loadings, random slopes (when the observed variables are categorical), and three-level latent variable models that have categorical variables (Muthén 2010). On the other hand, Bayesian estimation which is based on Markov chain Monte Carlo algorithms has proved its usefulness in models with nonlinear latent variables (Arminger and Muthén 1998) or multi-level latent variable factor models (Goldstein and Browne 2002), and those which can be generated on the basis of a semiparametric estimator (Yang and Dunson 2010). Moreover, Bayesian estimation has helped to obtain impossible parameter estimates, thus aiding model identification (Kim et al. 2013), producing more accurate parameter estimates (Depaoli 2013) and aiding situations in which only small sample sizes are available (Zhang et al. 2007; Kaplan and Depaoli 2013). Also, with the Bayesian approach to SEM, researchers may favorably present their empirical research, e.g., in the confidence interval (CI) (Scheines et al. 1999).

### Multi-level SEM modeling

Other progress that was made was the adaptation of multi-level analysis (MLM) in multi-level SEM regression modeling for latent variables (ML-SEM). The multi-level regression models were primarily used to secure consistent estimates of standard errors and to test statistics due to dependent observations within the clusters, which represented typical examples of data based on a hierarchical structure (e.g., individuals nested within households which are also nested within neighborhoods or districts). Much later, the logical next step was a general model for multi-level structural relations accommodating latent variables as well as the possibility of finding missing data at any level of that hierarchy.

Multi-level analysis was first proposed by Goldstein and McDonald (1988), McDonald and Goldstein (1989), Lee (1990) and McDonald (1993, 1994). However, it was Meredith and Tisak (1984) who had a ‘vision’ of combining SEM with MLM. The other works referred to problems associated with multi-level and multiple group structural equations analysis (McArdle and Hamagami 1996), the intersection between SEM and MLM using separate structures for fixed and random effects to make them maximally consistent (Rovine and Molenaar 2000, 2001), or the application of SEM in estimating MLM for dyadic data (Newsom 2002). Attention was also paid to problems connected with estimating both balanced and unbalanced designs for linear structural relations in two-level data.^7^ Muthén (1990, 1991, 1994) proposed, for example, a partial maximum likelihood solution as a simplification of the unbalanced designs, which entailed the computation of a single between-groups covariance matrix and an ad hoc estimator/scaling parameter. He also presented the way to estimate the two-level SEM by using available SEM software. Consequently, multi-level SEM models allowed researchers to create separate models for within-cluster and between-cluster covariance matrices (Matsueda 2012). Muthén’s proposal was grounded on the specification of separate within- and between-cluster models, and on further application of the multiple-group option to estimate the parameters simultaneously. Muthén (1994) argued that by using this method the estimator would behave equivalently to the maximum likelihood in balanced designs and would be consistent (with reasonable standard errors and test statistics) in unbalanced designs. Much later, Liang and Bentler (2004) debated on the similarities and differences between the various formulations of two-level structural equation models and presented a computationally efficient EM algorithm for obtaining ML estimates for unbalanced designs which included cases missing at random.

Further development of ML-SEM analysis can be dated to the work of Rabe-Hesketh et al. (2001, 2004) and Skrondal and Rabe-Hesketh (2004), who proposed a more general approach to multi-level structural equation modeling which, at present, is known under the name of Generalized Linear And Mixed Modeling (GLLAMM—computer software) and which is based on three related parts: the response model, the structural equation model for latent variables, and the distributional assumptions for latent variables. GLLAMM was prepared for a wide range of multilevel latent variable models that can be used for multivariate responses of mixed type, including continuous data, duration/survival data, dichotomous data, or ordered/unordered categorical responses and rankings. The opportunities of using GLLAMM range from multi-level generalized linear solutions or generalized linear mixed models, through multi-level factor or latent trait models, item response models, latent class models, to multi-level structural equation models.

### Meta-analysis in SEM

The necessity of comparison and synthesis of all research findings across the studies has laid the foundations for meta-analysis in SEM (MA-SEM), which was created on the basis of three main objectives: testing the consistency of the estimates and effect sizes in different studies, estimation of a polled effect size, and identification of potential moderators that influence the model’s structure (Viswesvaran and Ones 1995; Hunter and Schmidt 2004). Researchers can also use MA-SEM in testing causal models (e.g., Becker and Schram 1994; Miller and Pollock 1994; Shadish 1996).

Since the theoretical principles had already been worked out, both for MA and SEM, a natural step in further methodological work was to find answers as to how to secure the effective integrity of these two statistical approaches. It should also be mentioned that MA and SEM were developed under different research traditions, e.g., the statistical theories of MA and SEM were based on distributions of correlations and covariance matrices, respectively. Hence there is no guarantee that inferences based on combining these two approaches will always be correct. Moreover, empirical studies on the validity of these procedures are still rare (Cheung 2000, 2002; Hafdahl 2001).

A first assumption that was stated in the literature in the context of combining SEM with MA was fitting the structural equation model on the meta-analyzed covariance or correlation matrix. Subsequently, two (often complementary) approaches were proposed based on univariate and multivariate methods. In the former approach, which is similar to conventional meta-analysis, the class of univariate methods refers to the elements of a correlation matrix which are treated as independent within studies and are pooled separately across studies (e.g., Brown and Peterson 1993). The dependency of correlation coefficients (calculated as untransformed correlation coefficients *r* or transformed coefficients *z* in meta-analysis, e.g., see the works of Corey et al. 1998; Hunter and Schmidt 1990; Schulze 2004; Cheung and Chan 2005; Furlow and Beretvas 2005) within the studies is not taken into account (as opposed to multivariate methods), and a population value is estimated for each correlation coefficient separately. Later, when the correlation coefficients are pooled across studies (using the *r* or *z* method), one pooled correlation matrix can typically be constructed from separate coefficients, and the hypothesized structural model can then be fit into this matrix as if it were an observed matrix in a sample. Thus the main problems of the univariate methods as referred to in the early development of MA-SEM were: the lack of dependency of the correlation coefficients; non-positive definitive correlation matrices (Wothke 1993) due to different elements of the matrix which are based on different samples; lower level of precision in the correlation coefficients; and different results for different sample sizes which are associated with different correlation coefficients.

In the case of the latter approach, i.e., the multivariate method, two strategies were proposed, i.e., generalized least squares (Becker 1992) and two-stage structural equation modeling (Cheung 2002; Cheung and Chan 2005). Becker (1992) used GLS estimation to pool correlation matrices by taking the dependencies between the correlations into account. That meant not only that the sampling variances but also the sampling covariances in each study could be used to weight the correlation coefficients. Later, as the population parameters become unknown, estimates of the covariances between correlations can be obtained by plugging in sample correlations. However, because estimates from a single study are often not stable, Becker and Fahrbach (1994) and Furlow and Beretvas (2005) recommended that pooled estimates of population correlation be used by using the weighted mean correlation across samples. In contrast, in the two-stage structural equation modeling that was proposed by Cheung (2002) and Cheung and Chan (2005), multi-group SEM could be applied to pool the correlation coefficients at stage one and then in stage two, and the structural model could be fitted to the pooled correlation matrix by using weighted least squares, i.e., the WLS estimator, in which the weight matrix in the WLS procedure represents the inversed matrix with the asymptotic variances and covariances of the pooled correlation coefficients from stage one. This ensured that correlation coefficients which were estimated with more precision (based on more studies) in stage one obtained more weight in the estimation of the model parameters in stage two. However, the precision of a stage-one estimate depended on the number and size of the studies that had reported the specific correlation coefficient.

To sum up, the MA-SEM has introduced important quality to SEM by providing an integrative framework analysis in which researchers can pose scientific questions that are not necessarily addressed in one study. Researchers have gained the opportunity to test structural models that have not been tested in any primary study (Viswesvaran and Ones 1995). This is a truly attractive feature of the MA-SEM because one can empirically test the viability of a structural model (or perhaps of competing models) by combining the available evidence from potentially disparate literatures. Second, as noted in treatments of SEM (e.g., Barrett 2007; Kline 2011), strong inferences from tests of structural models are dependent on a sufficiently large sample (i.e., at least 200 cases). Although SEM studies can certainly achieve reasonable sample sizes, meta-analytic correlations are typically generated from samples that far exceed these minimum values, which means that the parameter estimates and fit statistics will be more stable than values generated from any single sample (i.e., primary study) (Landis 2013). Also, the MA-SEM was initiated for testing hypothetical models across studies with the same meaning, research context, and with the purpose of a comparison of these models (MaCallum and Austin 2000).

### Multi-group SEM analysis

Besides the possibility of comparing research findings in the MA-SEM strategy, the necessity of diagnosing group/sample similarities based on multi-group SEM analysis (MG-SEM) also appeared in the literature. To recall, in the social sciences researchers often have to deal with several samples-groups arising from one or several populations. Consequently, it is important to understand to what extent these might differ (Muthén 1989a, b). In SEM this can partially be achieved by testing the equivalence of the SEM model parameters, such as factor loadings, path coefficients, or variances of factors (Yuan and Bentler 2001).

MG-SEM was initiated by Jöreskog (1971), who developed a maximum likelihood estimator for SEM with multiple groups, and by Sörbom (1974), who studied differences in the context of factor means across groups. Sörbom’s approach later became a generally accepted solution thanks to the work of Browne and Arminger (1995), who renamed it the Mean And Covariance Structure—MACS. Next, Bentler et al. (1987), and later Muthén (1989a), proposed an alternative approach, i.e., the generalized least squares estimator, and Satorra and Bentler (2001) developed scaled tests in the multi-group analysis of moment structures. Finally, Muthén (1989b), Yuan and Bentler (2001) worked out solutions which helped to eliminate problems with the so-called nonstandard samples (e.g., containing missing data, nonnormal data, and data with outliers).

The process of MG-SEM analysis entails that some path coefficients in a multi-group SEM model are constrained equally, while other coefficients remain varying, across groups. By testing the equality or invariance of path coefficients across groups, the researcher obtains the opportunity to examine whether different groups behave similarly (Hayduk 1987). Initially, when the population covariance matrices are deemed to be equal across groups, the next step to substantiate the measurement invariance is to check whether the sample covariance matrices in all of the groups can be adequately fitted by the same model. Then the cross-group equalities of factor loadings, error variances, factor variances-covariances and structural paths are taken into account sequentially. We also assume the mean structures, where cross-group equalities of intercepts and factor means are examined. Usually, the golden rule of MG-SEM analysis states that if the statistic at the hypothesized model is not significant at a level of 0.05, the researcher can move to the next level by using a Chi-square-difference statistic. However, as Yuan and Chan (2016) and Yuan et al. (2016) argued, this rule is unable to control either type I or type II errors. Therefore, to overcome this problem they suggested to modify the process of testing the null hypothesis via equivalence testing, which allows researchers to effectively control the size of the misspecification before moving on to testing a more restricted model.

### Latent growth curve modeling

Finally, the last milestone in structural equation modeling was latent growth curve modeling (LGCM). In LGCM the analysis is based on repeated measures, in which the latent variables are conceptualized as aspects of change and the factor loadings are interpreted as parameters representing the dependence of the repeated measures on these unobservable aspects of change (see the classic works on the subject written by Meredith and Tisak 1990; McArdle and Epstein 1987; Willett and Sayer 1994; Raudenbush 2001; Bollen and Curran 2006; Duncan et al. 2006; Preacher et al. 2008). Because time is an indicator of the modeled trends, in LGCM we model the longitudinal data, in which repeated measurements are observed for some outcome variable, at a number of occasions.

The roots of LGCM analysis can be found in panel data modeling (see the comments by Tucker 1958; Rao 1958); however, it was Meredith and Tisak (1990) who published the treatment within an SEM framework that is still relevant today. Rao and Tucker constructed a procedure that included unspecified longitudinal curves or functions, while Meredith and Tisak showed that individual growth curves, which are often modeled within a multi-level or mixed model framework (Raudenbush and Bryk 2002), can be modeled within a standard SEM framework by treating the shape of the growth curves as latent variables with multiple indicators at multiple time points. Other directions of LGCM development were continued in the area in which time points, or the spacing between time points, could vary across individuals (Hui and Berger 1983; Bryk and Raudenbush 1987). Thus latent growth models could be applied to circumstances in which individuals were not measured within the same intervals. Consequently, latent growth models can be imbedded in many theoretical models, thus allowing for a more comprehensive and flexible approach to research design and data analysis than any other single statistical model for longitudinal data in standard use in the social sciences. As Duncan et al. (2006, p. 4) commented , “some of the strengths of these models include the capacity to test the adequacy of the hypothesized growth form, to incorporate time-varying covariates, and to develop from the data a common developmental trajectory, thus ruling out cohort effects”. Furthermore, it should be mentioned that SEM can be applied to longitudinal data not only in the form of latent growth curve models but also in the form of autoregressive models and stochastic differential equations (e.g., Oud and Voelkle 2014; Voelkle 2008; Voelkle et al. 2012). This development even allows to treat SEM as a more general analytical strategy.

Finally, the hybrid of the latent growth and latent class models should be mentioned that was proposed in 1993 by Nagin and Land, who developed a model based on the group trajectory. This configuration of both models allows to estimate individual trajectories by using polynomials and then to classify these trajectories into discrete groups. The latent classes can be viewed as points of support in approximating a continuous distribution of the unobserved heterogeneity or as reflections of theoretically important groups. Simultaneously, Muthén (2004) developed growth mixture modeling with which researchers are able to analyze within-class variation among individual trajectories (a mean curve with variation around it).

## General statistical and philosophical controversies regarding the use of SEM models in the social sciences

In the dynamic development of SEM, statistical and philosophical controversies were also present in the use of this analytical strategy. The statistical aspects mainly refer to specific technical-methodological problems, whereas the philosophical aspects refer mainly to the ontological nature of causality and to the role of SEM in the epistemology of causal inference on the basis of experimental or nonexperimental data. All in all, the practical issues of using SEM models in research can be boiled down to difficulties in the application of such types of models because of their high level of complexity (see e.g., Bagozzi 1983a; Breckler 1990; Cliff 1983; Fornell 1983; Tomarken and Weller 2003). Below we will explain in detail some of the problems and controversies pertaining to the construction and application of SEM models.

### Problems in understanding the role of the null hypothesis and equivalence in SEM models

One of the main difficulties in using SEM is the reversed role of the null hypothesis which complicates the process of statistical inference, particularly in the course of interpretation of the model results. Yet another factor that influences SEM is the ontological character of the validity of the tested model. We assume that the tested model is exactly true when testing the overall fit of an SEM model; however, such a model only approximately represents substantive processes, which means that the hypothesis explaining the exact goodness of fit of the model is a priori false. SEM models represent only approximate estimates of the substantive processes. Consequently, the only realistic hypothesis that might be assumed is the hypothesis of the near/close fit (Cudeck and Henly 1991; MacCallum and Tucker 1991). Browne and Cudeck (1993), conducting their studies based on the work of Linhart and Zucchini (1986), presented even conceptual and mathematical strategies allowing for the representation and evaluation of various sources of errors in the relationship between models and empirical processes. Their work allowed for the formal identification of many critical aspects of testing SEM models, such as test of close fit (Browne and Cudeck 1993), cross validation (Browne 2000a), and statistical power analysis (MacCallum et al. 1996).

Moreover, factors which complicate inference from SEM refer to the problem of equivalence. To recall, equivalent models represent various patterns of relationships between variables but also have the same number of free parameters (and degrees of freedom) and the same statistical values of the model fit. Thus equivalent models are hardly ever comparable only on the basis of statistical criteria. MaaCallum et al. (1993) demonstrated that for each substantive model one can actually create one or more equivalent models; therefore, models of this kind complicate quality assessment in SEM. Regardless of the adequacy of the proposed theoretical model and the level attained, other alternative models may have an equivalent degree of fit to the sample matrix **S**. Consequently, any failure to reject the proposed model does not automatically mean confirmation of the veracity of that model (Cliff 1983; James et al. 1982). In other words, the presence of equivalent models reminds us that although an inadequate fit of the model to the data implies the failure of the proposed theoretical model, an adequate fit does not necessarily confirm the validity of the proposed substantive process. A truly valid model of the substantive process reflects just one of many possible models which have an adequate fit to the data.

### Difficulties in the specification and modification of SEM models

Another source of controversy is inappropriate specification and modification of the SEM model. The idea of a ‘specification search’ was discussed by MacCallum (1986) but was originally described by Leamer (1978) as the process of modifying the model in order to either simplify or improve its fit to the empirical data. Thus, modification seems to be necessary for all SEM models because they rarely pass the test of fit when compared with the set of data in the first stage.

There are two general types of specification errors. The first error can be caused by omission, while the second is based on inclusion of more parameters in the SEM model than it requires. In order to eliminate these errors, researchers are allowed to use the strategy of a ‘forward’ or ‘backward’ specification search. The forward specification corrects any omissions of parameters. The process usually starts with a more restricted model and then leads towards a more general model (Bentler and Chou 1993; Chou and Bentler 1990). Errors are usually detected by LM (Lagrange Multiplier) tests or the EPC (Expected Parameter Change) index (see e.g., Bollen 1989a). On the other hand, the backward strategy corrects any possible errors of inclusion. The process begins with a more general model and continues towards a more restricted model which is verified with the W (Wald) test.

A problematic issue with the above strategies is that there is no literature consensus as to which is the best strategy. In particular, this lack of agreement is visible when discussing the model’s errors, sample errors, or violations of normality in the variables (Green et al. 1999; MacCallum 1990; Hayduk 1990). Another problem is that a model which is theoretically too complex is deprived of the basics in substantive theory. As a result, and as Wheaton (1988) argued, SEM models always undergo modification to improve their fit to the data, which also means that every model becomes empirically determined and simultaneously loses its status of an a priori hypothesis. Bollen (1989b) even claimed that modification in the initial model leads to a purely exploratory analysis; therefore, the probability levels of tests related to statistical significance for new (modified) versions of the models should be regarded as only approximations (Bollen 1989b). Also Byrne (2010, p. 89) explained that “once a hypothesized model has been rejected, this spells the end of the SEM as the analytic approach, in its true sense. Although SEM procedures continue to be used in any respecification and re-estimation of the model, these analyses are exploratory in the sense that they focus on the detection of misfitting parameters in the originally hypothesized models”. Thus, any changes ever made to the model are actually data-driven and lead the researcher from the realm of hypothesis testing or confirmatory analysis to the domain of exploratory analysis. On the other hand, Arbuckle (2007, p. 234) added that “in trying to improve a model the researcher should not be guided exclusively by modification requirements. A modification should be considered only if it makes theoretical or common sense”. If it is not, such a strategy is prone, through capitalization on chance, to produce absurd models with satisfying Chi-square values but which are deprived of theoretical sense. These issues were discussed extensively by MacCallum (1986) and MacCallum et al. (1992).

### Errors caused by omission of important variables in SEM models

The practical implications of using SEM models also lead to problems regarding the omission of important variables in the model. The selection of variables comprises a critical element of SEM model construction since the errors of omission of important variables (both exogenous and endogenous) have an influence on every parameter of the tested SEM model. Just as in the case of omitting particular parameters, the omission of important variables leads to biased parameter estimation and incorrect standard errors.

The issue of omitting important variables in SEM was described generally from the perspective of unmeasured variables (James 1980; Sackett et al. 2003; Tomarken and Weller 2003, 2005), however, Cohen and Cohen (1983) have also researched the issue from the perspective of obtaining spurious relationships. Finally, James et al. (1982) and Bollen (1989a) addressed this issue from the perspective of the self-containment violation or pseudo-isolation of the equation system for causal inference. In the assumptions underlying pseudo-isolation, researchers may believe that each equation of the examined model possesses a sufficiently large number of important exogenous variables and that the residuals in the equations include only random disturbances that are not correlated with exogenous variables. As we know, biases in model parameters caused by the omission of important observable variables (exogenous or endogenous) apply to all tested types of SEM models. Thus the problem of pseudo-isolation is a serious threat to causal inference in SEM analysis.

Given the above, James (1980) pointed out that the relevant questions posed by researchers should not refer to the issue of whether there is a problem in omitting variables but about what the magnitude is due to the consequences this problem has on the estimated parameters in the hypothesized model. Unfortunately, there are no simple mechanisms which might help to accurately detect that magnitude. This problem still remains unsolved, though there are two forms that this issue can be addressed by. The first approach is called the test of an incorrectly specified model (Arminger and Schoenberg 1989) and was adopted from Hausman’s test (1978), while the second approach relies on sensitivity analysis. The problem with Hausman’s test lies in its high sensitivity level that is caused by a correlation between residuals in the equations and the exogenous variables. Therefore, although Hausman’s idea (1978) is, on an intuitive level, relatively simple to understand, in practical terms its implementation will be complicated. Besides, the test has two limitations: first, it is sensitive to violation of normality distribution of the residuals; second, simulation studies have showed (Long and Trivedi 1993) that Hausman’s test does not provide the model’s optimal properties for small sample sizes.

The second approach, i.e. sensitivity analysis, was created by Rosenbaum (1986) and further developed by Mauro (1990) and Frank (2000) in estimating the structural path coefficients, assuming various levels in the values of correlations (theoretically probable and empirically acceptable) between the exogenous variable (omitted in the model), and variables (endogenous and exogenous) which were already included. A direct transposition of the sensitivity analysis from the regression model on SEM might be, however, challenging for a researcher who might be ready to use this method but may not know the methodological nuances. Actually, a sensitivity analysis assumes knowledge not just about the omitted variables in the model but also about the relationships of these variables with other variables in the model. To sum up, the effects of omitting an important variable in the SEM model (either exogenous or endogenous) depend on the form of the tested SEM models, the role of the omitted variable in the model, and the pattern of relationships between the excluded variables and the variables included in the model.

### Problems with multicollinearity in SEM

Another issue that largely complicates the inference process in SEM analysis is multicollinearity. The problem of multicollinearity stems from the inclusion of redundant variables in model, as these carry a limited level of information. In this sense, multicollinearity represents the inverse of problems pertaining to the omission of important variables in SEM analysis. However, similarly to omitting important variables in the model, multicollinearity can challenge the results of SEM analysis. In the context of SEM, as compared to regression analysis, multicollinearity results from too strong a relationship between observable data and a particular form of the tested model. Models strongly vulnerable to multicollinearity are those which propose moderating relationships (Little et al. 2007) as well as latent growth curve models (Biesanz et al. 2004).

Despite the potentially serious consequences for statistical inference, the problem of multicollinearity has been relatively little investigated in the context of SEM. Researchers have not paid sufficient attention to the possible consequences of multicollinearity on SEM (Grewal et al. 2004). In fact, this knowledge contrasts with advanced knowledge on regression models for which methods of detection and correction of multicollinearity based on the variance inflation factor (VIF) or variance-decomposition proportion (VDP) (see e.g., the works of Belsley 1991; Cohen and Cohen 1983; Draper and Smith 1981) have already been developed. Also, the approach to overcoming the problem of multicollinearity in SEM is far more difficult than in the case of classical regression models, e.g., in SEM the independent variables can be both exogenous *X* and endogenous *Y*, and in regression analysis the incorrect configuration of the variables simply refers to exogenous, i.e. independent variables *X*. Moreover, in SEM the variables, being in strong multicollinearity, can assume a latent character. Another difference refers to the relation between estimation of the model parameter variance and the independent variables that remain in a linear relationship. In the regression model, each independent variable is connected only with one estimation of the model parameter variance, whereas in the SEM model one independent variable can be connected with estimations of a few model parameter variances. This problem becomes even more complex when strong multicollinearity concerns two or more parameters which correlate latent variables.

The issue of multicollinearity in SEM models, particularly concerning the consequences of multicollinearity for statistical inference, can also be described in the context of empirical underidentification (Grewal et al. 2004).^8^ Kenny (1979) pointed to multicollinearity between observable exogenous variables of the **S** matrix as one of the main causes of empirical underidentification of the model. Also, Rindskopf (1984), Bollen (1989a), Kenny et al. (1998) developed the notion of empirical underidentification of the SEM model as an effect of multicollinearity between latent variables. On the other hand, Hayduk (1987) created a visual analysis of the covariance matrix of estimates pertaining to model parameters as a way to diagnose multicollinearity. Finally, Kaplan (1994) developed the approach of Hayduk (1987) by using dependency analysis of the correlation matrix based on SEM model parameter estimates. With this purpose in mind, he used the so-called conditioning number, conditioning index and decomposition of variances (originally applied to incorrect conditioning of the **X** ^T^ **X** matrix in regression models) as measures of incorrect conditioning of the correlation matrix of parameter estimates in the tested SEM model. These solutions were considered from the perspective of two primary levels. At the first level, the problem of multicollinearity was described in the context of an independency of the tested model and referred only to the domain of conditioning covariance matrix **S** for observable variables. At the second level, the multicollinearity problem was characterized as a dependent form from the tested model. Both forms of multicollinearity may be engaged in the fallacious process of inference associated with SEM, hence both require separate approaches to diagnose and correct the multicollinearity. Also, standard estimators such as ML, GLS, and ADF often require a positive-definitive **S** matrix. Consequently, extremely incorrect data conditioning, which creates the effect of singularity of the **S** matrix, disqualifies the results of the SEM analysis. Given this, Wothke (1993) proposed a method of diagnosing the poor conditioning of the **S** matrix on the basis of eigenvalues and eigenvectors. Another solution that was proposed in the case of singularity of the **S** matrix concerned the application of a ridge estimator (Jöreskog and Sorböm 1996) and a maximum entropy estimator.

Because there are no simple ways of correcting the problem of multicollinearity, the analytical strategies may refer to finding compromises between three elements, i.e., data, methods of parameter estimation, or the form of the tested model. When making a correction of the poor conditioning of the **S** matrix, one can extend the existing dataset with new units in the sample. However, this approach does not guarantee improvement and correct conditioning of the **S** matrix. On the other hand, incorrect conditioning can be regulated by deleting redundant observable variables. Unfortunately, this approach requires a re-specification of the SEM model. Simultaneously, poor conditioning of the **S** matrix can be overcome by implementing the ridge or ULS (unweighted least squares) estimators. Finally, the correction strategy can be boiled down to a reformulation of the tested model. In extreme cases, such a modification may lead to a change (reduction) in the number of latent variables, e.g., by investigating the discriminant validity between the latent variables, and in less extreme cases one can enter equality restrictions on the estimated parameters of the tested model which remain in a strong linear dependency.

### Relationships between model fit and strength of correlation of the observable variables

The last statistical problem to be discussed here which complicates inference on the basis of the SEM model is the negative relationship between measures expressing the general (absolute) fit of the model and the strength of the correlation of the observable variables in **S** (Fornell 1983; Fornell and Larcker 1981; Marsh et al. 2004a, b). To recall, a weak correlation between the observable variables increases the probability of achieving a good fit of the model. Fornell and Larcker (1981) pointed out, over a quarter of a century ago, that if the correlations of the observable variables are sufficiently weak, almost every (correct or incorrect) model will have an adequate level of the matrix fit measured with the use of the Chi-square statistic and of the derivative descriptive measures of a general fit. Simultaneously, Browne et al. (2002) pointed to such a dependency in the context of reliable measurements. The effect of this dependency can be a rejection of the correct model, e.g., when the level of model reliability is high, or acceptance of the incorrect model in conditions of low reliability of the measurement. Later, Tomarken and Weller (2003) stressed two discrepancies that appear between measures expressing the absolute fit and the statistics of the examined SEM model parameters. The first discrepancy is caused by a negative relationship between the level of measurement reliability and the fit of the model, while the second is caused by the positive relationship between measurement precision and estimation precision (which is expressed in lower standard errors) of the SEM model. In SEM, the former relationship is highly undesirable, while the latter option is highly expected.

To sum up, information about the relationship between measures of the general model fit and observable variables in the **S** matrix as well as the reliability indices carries tremendous implications for the practice of SEM model analysis. Being aware of these relationships requires us to make appropriate conclusions, e.g., whether the good fit of the model is not by any chance the result of low correlations between the observable variables considered in the model. Such conclusions can be conducted almost on the basis of the correlation **R** matrix before proceeding to a final interpretation of the model. Consequently, in SEM models with latent variables, low reliability will signal that, formally, a good fit of the tested model to the **S** matrix was probably obtained due to low correlations between the observable variables. The reverse situation is also possible, e.g., if the hypothesized model does not appropriately fit the **S** matrix but simultaneously obtains a higher level of reliability, one can conclude that the source of this mismatch is not the inadequacy of the model but a very good selection of the measurement instrument (Browne et al. 2002).

### Theoretical–philosophical controversies in the domain of SEM model usefulness

Apart from the technical issues as mentioned above, difficulties in using SEM models in the social sciences also appear in the domain of philosophical arguments. The main ontological critique of SEM is the latent reality in the social sciences which is so complex that even a simplified statistical model of this reality can be of no cognitive significance. On the other hand, the main epistemological argument states that the use of SEM with nonexperimental data only misleads researchers by creating the illusion of causal process modeling. We will discuss these problems in depth.

The ontological argument claims that it is impossible to transform the investigated phenomenon into a limited mathematical form (i.e., into a system of structural equations) which would justify certain regularities in social life. Moreover, even if such regularities exist, or processes of data generation are found, they are often doomed to be outdated soon after their identification because the availability of new sources of data outdates the theory. The advocates of ontological arguments (e.g., Cartwright 1999, 2001; Fox 1997; Rogosa 1987, 1988, 1995) pointed out that social reality in the world is so complicated and simultaneously ‘open’ to many changes that its representation in the form of mathematical rules is impossible, and that the statistical models constructed for such substantive processes can only be a simplification of this world. Cartwright believed that an essential element of SEM model construction is horizontal reductionism which limits, to a greater extent, the scope of possible applications of the model or its ‘validity’. Also, Fox (1997) pointed out that substantive theories in the social sciences are often vague, imprecise, and highly relative. As a result, any statistical binding of such theories with empirical reality is extremely difficult. Rogosa (1987, 1988, 1995) even stated that the causal SEM models have no scientific value because they represent models of relations between variables and do not represent causal processes at the level of the individual. Substantive processes take place in individuals and not between variables. Thus a model which ignores the individual process of data generation is of little substantive use.

However, when we consider the ontological assumptions, the argument of the openness of social life and the SEM model’s limitations are not new. In any case, this argument was the main source of disagreement regarding path analysis between Wright (1920) and Niles (1922, 1923). Wright (1920, p. 330) was fully aware that the path coefficients in a system of causes and effects can be calculated if a sufficient number of simultaneous equations can be made expressing the known correlations in terms of the unknown path coefficients and expressing the complete determination of the causes to effects. Niles (1922) believed, however, that the lack of profound knowledge regarding the mechanisms of the causal process eliminates the usefulness of path analysis. Wright (1920) accepted the human inability to acquire thorough knowledge regarding the causal process but, at the same time, he claimed that selective knowledge about the causal process allows one to isolate a part of this system and to calculate the relative correlation of the variables in this isolated part of a wider system.

Also, the presentation of other consequences related to SEM, including the specification of errors and consequences of omitting important variables, raises controversial issues which have an influence on the appropriate statistical inference conducted on the basis of SEM. However, we must not forget that all of these doubts can take on the form of extreme expansionism in either denying or questioning any attempt of description of the substantive processes analyzed from the perspective of statistical models such as SEM. We cannot forget that the exclusive knowledge of the correctness of the constructed model, or the inclusion of all necessary variables for the sake of the analyzed phenomenon, is possessed by a higher being only. Thus Glymour (1999), just as Wright (1920), and the vast majority of scholars (MaCallum 2003) were fully aware that SEM models are a priori at least to some extent imperfect but, on the other hand, they believed that valuable knowledge can be available through SEM, which, however, does not exempt one from striving to have full awareness of the limitations of such an analytical strategy.

Finally, the epistemological argument refers to the significance and role of SEM models in causal analysis. As Meehl and Waller (2002) noticed, some researchers (such as Blalock 1991; Heckman 2005; Irzik and Meyer 1987; Pearl 1998a, b, 2000) adopted the SEM strategy as the best way of causal inference with nonexperimental data, while others (e.g., Freedman 1987, 2006; Sobel 1995) thought that using SEM would be misleading because it gives a promise or an illusion of causal process modeling with nonexperimental data. The current consensus has been reached more in the direction of a careful application of SEM as an established method of investigating causal relationships from the perspective of nonexperimental data (see, for example, papers by McDonald 2004; Meehl and Waller 2002). These controversies surrounding the SEM role in causal inference should not be surprising because, as Cook and Campbell (1979) explained a long time ago, the epistemology of causal inference remains in a productive state of chaos. This view was also confirmed in a discussion between Cartwright (1989) and Glymour (1999) as well as between Humphreys and Freedman (1996), as it concerned the conditions enabling causal inference from passive observations.

Perhaps the most fervent critic of SEM models and their applications in causal analysis was Freedman (1987, 1991), who argued that SEM only distracts the researchers’ attention from real issues such as a strong substantive theory, an adequate research plan and an adequate process of data collection. The SEM, according to Freedman’s point of view, promises something that is actually unavailable in the process of applying a statistical model, as long as there exists limited knowledge on the subject of modeling substantive processes. Cartwright (1999) raised the same issue by claiming that causal processes are developed as the effect of multiple smaller causal mechanisms that cooperate and interact with one another. Knowledge concerning such types of mechanisms does not come from statistical data, and when this knowledge is already gathered, statistics soon become unimportant since the causal processes have been identified. Freedman (1987, 1991) also claimed that despite the statistical theory allowing to investigate the problems of measurement errors, new solutions based on nonlinearity,^9^ or the omission of important variables in SEM models, it does not enable one to test these problems and to completely eliminate them from SEM. Freedman (1991) indicated that the practice of making up for the restrictions of the substantive theory or the research plan and involving many competing models is also counterproductive. In response to these critics, Wright (1921, 1934) argued that path analysis was not expanded in order to discover the cause or causal relationships. Wright (1934) also pointed out that it is necessary to possess some knowledge about the substantive theory rather than to entirely rely on path analysis as a statistical method of generating this theory. Similarly, Duncan (1975) stated that no statistical method can be used as a final test of causal order and quoted the following words stated by Fisher (1970; p. 192): “if we choose a group of social phenomena with no antecedent knowledge of the causation or absence of causation among them, then the calculation of correlation coefficients, total or partial, will not advance us a step towards evaluating the importance of causes”. Finally, Pearl (1998a), when responding to Breckler (1990), again stressed that the knowledge or theory concerning causal relations is necessary because the observable relationships in a set of data cannot be the sole basis for causal inference.

Later, Freedman (1987) admitted that his arguments were based mainly on common sense, however, the world of science reacted to his critiques, claiming that most of the empirical applications in patch analysis have nothing to do with causal analysis, so the causality language must be replaced with a better language based on prediction (Muthén 1987b). There were even scholars (Bentler 1987) who claimed that Freedman’s critiques were simply the result of ignoring contemporary achievements that had been generated within this analytical strategy. However, despite all of these reactions, Freedman’s critiques (1987; 1991) became an important impulse for considering causal analysis and the role of statistical models in causal analysis in nonexperimental research (i.e., Heckman 2005; Cox and Wermuth 2004; Dawid 2000; Abbott 1998; Marini and Singer 1998; Sobel 1995, 1996, 2000; Pearl 2000, 2002; Holland 1986, 1988). As a consequence, causal analysis now covers two objectives. From one point of view it realizes the objective pertaining to some kind of activity (action) and thus supports social practice and diagnosis. From the other point of view, a causal analysis realizes cognitive objectives pertaining to scientific explanation. Holland (1986), Dawid (2000), Heckman (2005) and Cox and Wermuth (2004) indicated that these two areas of causal thinking require some kind of alternative approach or statistical treatment, e.g., obtaining action-oriented objectives requires thinking about the effects of causes or the magnitude of the causal effect, whereas obtaining the cognitive objective requires thinking about the causes of effects when we want to understand causal relationships between theoretical constructs. The analysis of the effects of causes is typically related to an analysis of counterfactuals (Holland 1986, 1988; Rosenbaum and Rubin 1983), whereas the analysis of the causes of effects refers to an analysis of the structural model (Cox and Wermuth 2004; Heckman 2005). However, up to now there has been no consensus as to which of these two objectives is possible to achieve through the postulate and test of the statistical model in the case of nonexperimental data. This discussion was continued by Holland (1988), who developed the approach of counterfactual states to the analysis of a simple recursive structural model, whereas Steyer (2005) presented concepts of individual and moderate causal effects which were central to the counterfactual approach within the SEM framework. Holland (1988) also promoted the issue that the variable gender or other factual variables which are not changeable cannot be introduced as causes to a model. This scholar argued that causes can only be variables which can be changed or manipulated. This was a valuable contribution to the development of SEM. Later, Pearl (1998a, 2000), starting from graph theory (Wright 1934), developed the theory of a Directed Acyclic Graph (DAG), which connected the counterfactual approach with the SEM model in a more common theoretical framework. These and other solutions in SEM made it possible to understand the meaning (the interpretation) of equations and structural coefficients of the model and allowed to determine the conditions within which the equations and coefficients may assume a causal interpretation. However, we also need to be cautious of the results of SEM analysis which can often be empirically overweighted. Consequently, if the hypothesized model is only empirically determined, at minimal theory input, then such a model is simply a convenient summary of a covariance matrix. The interpretation of this model’s equations expresses only the statistical relationships between variables in reference to the analyzed set of data. If, however, the tested SEM model is theoretically determined by a strong theory, then it receives the status of representing stable causal relationships in the empirical process of data processing. But even when taking these arguments into account we must still admit that in the literature there is no explicit consensus regarding the matter of determining the exact conditions allowing for causal analysis in SEM. This controversy became the main reason for many divisions between researchers who decided to implement the inductive approach in SEM (e.g., Scheines et al. 1994), those who claimed that causal analysis in SEM can be conducted only on theoretical foundations if they are supported by additional conditions (e.g., Cox and Wermuth 2004; Heckman 2005; Kenny 1979; Pearl 1998a), and those who simply rejected any possibility of analyzing causal processes in the form of the SEM model, particularly in reference to nonexperimental data (e.g., Freedman 1997; Holland 1995).

For example, inductive algorithms dedicated to discovering causal relationships were developed by Glymour et al. (1987) and further improved by Scheines et al. (1994), (1997), as well as Spirtes et al. (1993, 1995). Glymour et al. (1987), instead of focusing on the estimation and testing of structural models specified a priori, worked on computer algorithms (TETRAD) derived from artificial intelligence in order to ‘discover’ the causal structure. Thus they returned to the earlier ideas of Spearman, Frisch, Simon, Blalock, and Costner, who tried, in various ways, to induce a causal structure from patterns of association among variables (see Matsueda 2012). The basis of the TETRAD project was the belief that the observed correlations between variables might represent manifestations of a deeper sense, namely of the causal processes underlying the statistical relationships. The objective of TETRAD was thus not a criticism of the axiom explaining that the correlation of variables does not imply a causal relationship between these variables, as researchers involved in TETRAD questioned only the assumption that if causality between two variables would fail, then the possibility of finding any causality in the system of variables (e.g., with more than two variables) would also fail. However, despite the fact that the joint distribution of two variables does not provide causal information, a similar distribution including a system of many variables does indeed carry such information. This information can be detected on the basis of TETRAD inductive algorithms, where ‘causal independence’ (the causal Markov condition or the Reichenbach principle) and ‘faithfulness’ comprise the main assumptions underlying TETRAD, i.e. they bridge the statistical information and causality (Woodward 1998). A paradox, however, lies in the fact that the TETRAD project was also criticized (Cartwright 1989; Freedman 1997; Humphreys and Freedman 1996) for a severe inductive approach to analysis or, rather, the discovery of causal processes. Again, Cartwright (1989) argued that there is no possibility of discovering causal relationships on purely statistical grounds.

TETRAD was not, however, the main topic of discussion that was conducted at that time. Members of the SEM community were searching not only for alternative approaches to solve problems with causality in SEM but also slowly retreated from making rigorous causal claims and using causal language by proposing a strategy based on counterfactual thinking, e.g., “what would happen if a subject would receive a different treatment or value of the independent variable” (Cartwright 1995; Bollen 1989a; James et al. 1982). Consequently, the issues being discussed went further in the direction of the experimental and nonexperimental domain of data analysis. Particularly the experimental plans in SEM causal analysis provided important premises for detecting plausible causal relationships. Thus ‘manipulation’ and ‘randomization’ obtained a privilege in causal inference from data collected on the basis of experimental research.

In manipulation, the causal relationship represents a nonsymmetrical direction in which the assumption of time preference has to appear (Collingwood 1940). This assumption originates from the early works of Hume (1739), who claimed that the cause precedes the effect in time, although during SEM development this assumption has slightly been loosened. A good example might be a nonrecursive relationship in which casual asymmetry is not explained by time asymmetry. In most cases, however, researchers assume that (Bollen 1989a; Kenny 1979) a causal interpretation of the model will be stronger provided that the time preference will be demonstrated. In manipulation, the effect of the cause can be measured on account of the initiated changes, so in every case one needs to carefully design the research plans (e.g., in experimental or quasi-experimental research). In contrast, in nonexperimental research only a “passive observation between the state *X* and state *Y*, hence the causal relationship can be only observed on the basis substantive theory, nothing else” appears.

In the latter case, i.e., in randomization, Sobel (1995) and Heckman (2005, 2008) pointed out the differences between the so-called *fixing* and *conditioning* of exogenous variables as a key to distinguish real and seemingly causal effects. To recall, defining the exogenous variable is characteristic of an experimental situation in which the study’s participants are randomly selected and ascribed to levels of one or more exogenous variable(s). This fixing provides the credibility of the assumption that the selection which was conducted to a particular level of the *X* exogenous variable will be independent of the *Y* endogenous variable. Also, a fixing generates an effect where the exogenous variables are neither correlated with other exogenous variables nor with any prospective and unobservable causes (both the endogenous and exogenous variables). Since in nonexperimental research the process of selecting the random variable is rarely randomized, researchers have focused on the *conditioning* mechanisms. In research in which the process of selection is not random (quasi-experiments and nonexperiments), the identification of important correlates of this selection constitutes a cornerstone for conducting valid causal inference. It has been stated that a good causal SEM model should include at least two kinds of exogenous variables: *X* of which the causal effects remain in the area of substantive theory and variables *Z* which represent correlates of the selection process. Cox and Wermuth (2004) suggested that the adequate development of a causal process by the hypothesized SEM model sometimes requires that the researcher retreat causes *X* as endogenous variables which can be explained along with effects *Y* by the set of correlates *Z.* 10

Pearl (2000, 2002) presented analogous consequences of randomization and conditioning in the context of nonlinear causal models. His proposition of a nonparametric structural causal model could also hold regardless of the distributional and other statistical assumptions about a particular dataset. Hence, Pearl’s idea permitted the estimation of total, direct, and indirect effects without making any commitment to the form of the equations or to the distributions of the error terms, and it enabled an extension of the mediation analysis to systems involving categorical variables in the presence of nonlinear interactions.

Finally, Pearl (2000, 2002), on issues regarding causality, defined the causal assumptions on the basis of a graphical model, which has allowed many generations of researchers to logically infer the causality from a set of theorems applied to the graph. Pearl advanced SEM (see Matsueda 2012) in five general steps by: (1) the application of new mathematical notation to reflect causality, such as replacing the algebraic ‘equals’ sign with a sign that reflects a causal path; (2) deriving a theorem, i.e., the “back-door” criterion, to determine which covariates should be controlled to arrive at a causal relationship in the SEM; (3) deriving a theorem, termed “d-separation” (directed separation), which gives the necessary and sufficient conditions for independence between two sets of variables conditioned on a third set within an acyclic directed graph; (4) providing some simple mathematical notation to make counterfactual statements which can be analyzed within the directed graph; and (5) providing an algorithm for identifying equivalent models.

## Opportunities and threats in the application of SEM models in the social sciences: conclusions

Given the above arguments and facts, we should now conclude that the increase in the number of publications regarding structural equation modeling means that SEM is currently one of the most discernible analytical strategies in the literature that is being developed across many fields of the social sciences. The directions of all advances probably vary according to the substantive problems which individuals face in various disciplines. Thus SEM models can be extensively applied in the analyses of many processes and phenomena occurring, for example, in sociology, pedagogy, social policy, and the family sciences. They can also, which is extremely important, be applied in explaining economic phenomena, particularly in economy (see papers by Haavelmo 1943; Epstein 1987; Aldrich 1989; Golob 2003; Rao and Holt 2005) or in marketing research (e.g., Fornell 1983; Anderson and Gerbing 1988; Bagozzi 1980; 1983b, Bagozzi and Yi 1988; Hulland et al. 1996; Baumgartner and Homburg 1996; Chin et al. 2008). The generalizations of the SEM model mentioned in this article have also provided a basis for its broad use in psychology (e.g., Bentler and Speckart 1981; Kohn and Schooler 1982; Bynner and Romney 1986; Chassin et al. 1993; MaCallum and Austin 2000; Karimi and Meyer 2014), demographic research (e.g., Beckman et al. 1983; Thompson 1983; Linver et al. 2002), biology (Shipley 2000; Pugesek et al. 2003), genetics (e.g., Li 1956; Evans et al. 2002; Liu et al. 2008), or even in the medical sciences (e.g., Wheaton 1978, 1985; Chen and Land 1986; Bentler and Stein 1992; Skrondal and Rabe-Hesketh 2004; Raina et al. 2005; Stephenson et al. 2006) and in criminology (Matsueda 1982; Matsueda and Heimer 1987; Gibbs et al. 2003; Gau 2010). In short, the SEM approach, through its generality, can prospectively be applied in many different sciences; however, this does not yet mean that structural equation modeling is indeed applied by scholars in particular areas of the above-mentioned sciences. In fact, a recently published work by Saris and Revilla (2016) showed that in major journals in sociology, political science, and marketing, hardly any of the considered analytical models (which included latent variables) paid attention to issues of correction of the measurement errors, though these are the main reasons in the application of the SEM approach.

Admittedly, the advantage of SEM models over other approaches and statistical models is, above all, the fact that SEM allows to conduct a complex, multidimensional, and more precise analysis of empirical data taking into account different aspects of the examined reality and abstract concepts or theoretical constructs. To provide a simple example here, we can consider a theoretical construct measuring human intelligence which is not analyzed directly as it is in case of height or weight, but only through instruments designed to measure latent variables. Another good example can be studies related to marketing, in which SEM models provide explanations for constructs such as satisfaction and customer loyalty, or studies referring to relationships between shoppers’ personal values and their hedonic purchase behaviors (Steenkamp and Baumgartner 2000). Also, in management, SEM models (Shook et al. 2004; Marcoulides 2013) have proved their usefulness in examining, for example, relationships between market orientation strategies employed by companies or by the learning organization and attitudes expressed by the staff towards market research effectiveness (Tarka 2017). Thus analyses which are based on SEM offer more than the classical methods known in statistics. More importantly, the generality of SEM can be evidenced in the ability to parameterize the SEM to estimate a well-known class of alternative approaches known as general linear modeling (GLM) which includes, among others, the *t* test, ANOVA, ANCOVA, MANOVA, MANCOVA, or multiple regression. The SEM can be expanded to estimate measurement errors through the use of multiple indicator latent factors, the testing of complex mediational mechanisms through the decomposition of effects, and the testing of moderational mechanisms through estimation of multiple group analysis, just to name a few. Thus any linear model (e.g., regression) seems to be slightly worse as compared to SEM, not only due to the omission of correction in the measurement errors but also because of the fact that it is possible to ignore the indirect effects. Unquestionably, SEM is a significant and indispensable tool for empirical researchers. As Fornell (1983) argued, SEM reflects the second generation of statistical techniques that can be used to test the extent to which the respective research meets the recognized standards for high quality statistical analysis (Cook and Campbell 1979). In the first generation of techniques, Fornell (1983) included, among others, analytical methods such as multidimensional scaling, exploratory factor analysis, canonical analysis, variance analysis, or regression analysis, claiming that the advantage of SEM mainly consists in the possibility of identifying exact measurement errors during analysis, using abstract, non-observable constructs in the examined reality, analyzing their correlations, and confronting the given theory with the obtained empirical data. Thus SEM allows researchers to answer a set of interrelated questions in a single, systematic, and comprehensive analysis by modeling the relationships among multiple independent and dependent theoretical constructs simultaneously (Gerbing and Anderson 1988 **)**. This capability for simultaneous analysis greatly differs from most first generation statistical models (e.g., linear regression, LOGIT, ANOVA, MANOVA) which analyze only one layer of the linkages between independent and dependent variables at a time. Also classical statistical models, such as variance analysis, canonical analysis, or even discriminant analysis, make it only possible to conduct measurement on the basis of a single linkage between sets of dependent and independent variables, whereas structural modeling (SEM) makes it possible to simultaneously analyze data sets with many series of different linkages. Consequently, SEM allows for complicated variable relationships to be expressed through hierarchical or non-hierarchical and recursive or non-recursive structural equations, and to present a more complete picture of the entire model (Hanushek and Jackson 1977). Moreover, as Bagozzi (1980) claimed, SEM makes researchers define their research problems more precisely with reference to the examined phenomenon, makes them formulate clear targets and research hypotheses before starting the analyses, makes them provide precise definitions of the considered theoretical constructs and their further operationalization, and forces them to find the right, logic relationships between the constructs. Also, intricate causal networks enable SEM to characterize real-world processes better than simple correlation-based models. Therefore, SEM is more suited for the mathematical modeling of complex processes to serve both the theory and practice. SEM also provides a much better formal way not only to verify a given theory but also to conduct this verification on the basis of the analyzed measures, and because they join and at the same time confront the two spaces, i.e., theory and empirical data, they offer researchers a huge potential in the scientific explanation of phenomena alongside simple descriptive statistics or simple empirical relationships. As a consequence, researchers who choose the SEM model handle the analytical task with more precision than in the case of the procedure belonging to “traditional” statistics. The application of SEM analysis in the social sciences permits their development, because SEM, more effectively than ever, confronts the “theory” with “experience”, which results not only in optimizations of theoretical models but also leads to the optimization of tools used for the diagnosis of reality.

To sum up, researchers representing different fields of the social sciences can, or rather should, rely on SEM analysis and models in their research. The use of SEM will allow them to analyze complex research problems based on an analysis of cause-effect phenomena. It is the very complex structure of social phenomena that creates a growing demand for the use of SEM models, which are supposed to “copy” the social phenomena precisely. In other words, the researcher, when beginning to construct theoretical models which aspire to explain complex scientific problems of a social character, is, thanks to empirical verification, capable of gaining a better understanding of the importance of the examined phenomena. However, before the construction process of SEM models starts, the researcher should remember to maintain all theoretical assumptions in the SEM analysis. As it is known, in SEM the researcher should try, above all, to test the structure of covariance of the observable variables with the use of a simplified model structure and on the basis of the theory’s formalized elements. Thus SEM models are the synthesis of theoretical knowledge and current empirical results regarding the examined phenomena. The aim of the model construction is as simple an ‘explanation’ of the examined phenomena as possible, whereas the model itself should be coherent with the empirical observations. To recall, in science the explanation of an event is the logical deduction of that specific event from the true laws and background conditions surrounding the occurrence of that event (see Hempel 1965).^11^ Simultaneously, the process of model verification is, above all, a hypothetical-deductive process because the suggested model is constructed a priori and then confronted with the empirical set of observations. Scientists assess the validity of the hypothesis by empirically testing the other deductive consequences of that hypothesis.

Mulaik and James (1995, p. 118) once even stated that the “structural equation model as a representation of an objective state of affairs that stands in causal and/or criterial relationships with the data, and thereby as a causal explanation of the data, does not only reproduce the data. Something more is required with SEM: evidence supporting the assertion that the state of affairs represented by the model exists as represented, independently of the observer”. Structural equation models as mathematical models represent an objective state of affairs. In SEM analysis we do not make such assumptions that apply to statistical exploratory models, which means that we do not use the SEM model to find out something about the empirical data but, conversely, we use the given set of observations to verify the theory through the agency of the SEM model, or, actually, we try to explain to what extent the model reflects previous research or the theoretical assumptions which are the basis for the model’s construction (Kelloway 1998). That is why in SEM strategy researchers should not accept models at any price even when the tests used on them suggest that they should indeed do so. This would lead nowhere. SEM models are a kind of test and ‘indirect assistance’ which, on the basis of obtained empirical data and its verification, interferes with the whole process of explaining complex phenomena in light of a given theory. If the theory accepts rational justifications, the logic premises of a given phenomenon, and is, in other words, true, then it is the verification (with the use of SEM models) that should give evidence in the form of empirical results and, more specifically, we should express this evidence via the direction and values of the coefficients describing the relationships between the theoretical constructs which are considered in SEM analysis. On the contrary, if the model does not confirm the theory then we can draw two conclusions, i.e., proving that either the theory is wrong or that the material (empirical data) on the basis of which the SEM model was constructed is of poor quality.

At the same time, the fact that a given theory was confirmed by the SEM model does not yet mean that the theoretical assumptions should be considered as unquestionable, i.e., these assumptions only become more reliable and valid in light of the obtained results. They receive confirmation in the context of the conducted statistical analyses, on the basis of the adopted criteria in these analyses and of the levels of the theoretical assessment of the phenomena (Bollen and Long 1993). Therefore, a positive verification of the hypothetical relationships existing in the SEM model does not yet mean the end of the analyses, as we allow for the probability of building another theoretical model which can be far better than the previous one. What is more, for a given set of variables the researcher is usually able to determine many models representing completely different theoretical consequences but having an equivalent fit to the empirical data. Therefore, the theory of the examined process is whether a psychological, sociological, economic, or marketing background is a key criterion in choosing the model. Without a strong theoretical background or initial research conducted in the given field there is no way of being able to distinguish between many available ways to determine relationships between variables. In other words, it should be clear that a researcher cannot use SEM to derive a description of data such as “let the data speak for themselves”, because they do not, e.g., if the covariance matrix contains only 7 variables, one can sort these variables into 5040 different causal orders and therefore one can also obtain 5040 perfectly fitting saturated structural models, but one can also find many more alternative fitting models. That is why SEM can only be used given pre-specified assumptions about the order of the variables and the specific possible causal effects. What one virtually does with the SEM as an analytical strategy is to test if these assumptions are in agreement with the obtained empirical data. This is the only aspect that a researcher can do with SEM, therefore testing is crucial, which has already been proved in many works (see e.g., Mulaik et al. 1989; Bentler and Bonett 1980; Bentler 1990; Hu and Bentler 1999; Kenny and McCoach 2003; Hooper et al. 2008). In fact, researchers almost have an obsession over the global goodness of fit, i.e., the Chi-square test, because in large samples SEM models with many overidentifying restrictions tend to be rejected even when each restriction only departs trivially from the null hypothesis. This has caused a significant increase of other, alternative fit indices designed to offset the effect of sample size on test statistics. One of these was Steiger and Lind’s (1980) RMSEA, i.e., the root mean squared error of the approximation index, and Raftery’s (1993, 1995) application of Schwartz’s (1978) Bayesian Information Criterion index (BIC). For the former index, MacCallum et al. (1996) defined a noncentrality parameter which helped to calculate the power for the null hypothesis of a perfect as well as approximate fit. On the other hand, we must not forget that the major problem at that time was the power of the tests. Also, the sensitivity of fit indices to specification errors was not known. These issues were discussed by Satorra and Saris (1985) in the *Psychometrika* journal and by Bollen (1993). Satorra and Saris (1985) independently showed how to calculate the power of the likelihood ratio test in covariance structure models by using the noncentral Chi-square distribution and presented a nearly identical way of approximating the noncentrality parameter. They proved that the likelihood ratio test statistic is asymptotically equivalent to a quadratic form (Matsueda and Bielby 1986). Finally, in 1987 Satorra and Saris published, along with Dag Sörbom, a paper suggesting a different approach—the Expected Parameter Change (EPC) (Saris et al. 1987), while in 2009 they proved, together with Van der Veld, that the Chi-square test and the fit indices could not be trusted due to serious misspecifications which may not be detected and minor errors which may lead to a rejection of the model (Saris et al. 2009).

The issues which were discussed in this article reflect the fundamental problems which researchers may face in the analysis of complex social phenomena and structural equation modeling. Solving these problems, on the one hand, consists in optimization possibilities of theoretical models but, on the other hand, it comes down to a selection of appropriate technical tools in the diagnosis of the examined reality. SEM focuses namely on two sources, i.e., theoretical and technical. The most important sources are the theoretical premises from which we derive both the hypothesis about the measurement and the structural part of the SEM model. The hypotheses specify which parameters can obtain a given value (e.g., zero) and which parameters should be estimated on the basis of empirical data. The second source, in turn, does not result from the theoretical assumption of the examined phenomenon which the model should describe but from strictly technical reasons which, in most cases, focus on a description of parameter quality and model fit to the empirical data or on the description of relationships between measurement errors which occur naturally (or at least do not contradict the theory of the modeled phenomenon). However, in the end the basis for the evaluation and acceptance of the theoretical model, in which we assume certain hypothetical directions, is the positive result of the conducted procedure, which verifies the research hypothesis.

The technical issues of SEM became so unrealistic and complex that nobody pays much attention to the theoretical assumptions in SEM anymore, and technology tends to overshadow common sense (Freedman 1987). As a result, correct use of SEM is rare. The use of SEM has turned into a kind of fetish or numerical trick whose ultimate aim is to maximize the model fit to the data instead of using carefully differentiated research plans and careful substantive considerations on the grounds of theory. Steiger (2001) warned that this tendency has spread even in many published books which introduce SEM as an analytical strategy. Consequently, the authors of these publications are either unaware of the important technical issues of using SEM or they do not want to alienate the potential users of this analytical strategy. Even if the books carefully present the limitations of SEM, the practitioners have a prevailing tendency not to notice or even ignore these limitations. Hence the use of SEM in the social sciences is full of overuse, incorrect interpretation and overinterpretation. The risks are not posed by the SEM model itself but by the researchers’ lack of awareness of the SEM model’s complexity and limitations.

With reference to all of the above-mentioned advantages and disadvantages of SEM models and their application in the context of the social sciences, there are only two possible answers. We can either stop using SEM or work on the improvement of the practices of using this analytical strategy. Calling for an end to the use of SEM has little chance of gaining positive feedback from researchers, as SEM is too valuable and we cannot reject it. It seems that the only correct solution is to work on improving the use of this analytical strategy and obtaining an appropriate reference to it from the perspective of the analyzed data and the considered theories. As a result of these actions, SEM models will lead to better cognitive conclusions and correct results on the basis of the conducted empirical research.

## Footnotes

1 Wright’s publication released in (1918) referred to issues of modeling the size of animal (rabbit) bones.

2 Wright and other statisticians attempted to promote path analysis methods at Cowles (then the University of Chicago). However, statisticians at the University of Chicago identified many faults with path analysis applications to the social sciences—faults which did not pose any significant problems for identifying gene transmission in Wright's context but which made path methods problematic in the social sciences. Wright’s path analysis never gained a large following among US econometricians, but it was successful in influencing Hermann Wold (1956) and his student Karl Jöreskog.

3 Linear causal analysis had been introduced in sociology about one year earlier by the European scholar Boudon (1965).

4 The two-stage least squares algorithm was originally proposed by Theil (1953a, b) and more or less independently by Basmann (1957) and Sargan (1958) as a method of estimating the parameters of a single structural equation in a system of linear simultaneous equations.

5 Traces of polychoric and polyserial correlations can even be found in the works of Pearson (1901b) and Olsson (1979) as well as of Poon and Lee (1987), who worked on multivariate ML estimators both for polychoric and polyserial correlations.

6 There seems to be a growing consensus that the best contribution of Muthén to the analysis of categorical variables (especially with few categories) was the WLSMV or WLSM estimator implemented in Mplus (Muthén et al. 1997; Muthén and Muthén 1998) which adjusts the Chi-square statistic and standard errors by variance and/or mean. On the other hand, in LISREL and EQS a similar approach uses WLS together with polychoric correlations and asymptotic covariance matrices.

7 The use of multi-level SEM models in the early years was slightly limited since these models were hampered by the fact that closed-form mathematical expressions have only been available for perfectly balanced designs. Hence alternative numerical procedures were developed (e.g., iterative estimation procedures such as full maximum likelihood—Goldstein 1986; Longford 1987; restricted maximum likelihood—Mason et al. 1983; or Bayesian estimation—Kaplan and Depaoli 2013) in order to obtain efficient estimates for unbalanced designs (see e.g., Goldstein and McDonald 1988; Muthén 1997; Yuan and Bentler 2007). Most of these works focused on expanding SEM for application with dependent data structures (a violation of the assumption of independence), which often led to biased test statistics, standard errors, and even parameter estimates due to inappropriate aggregation across levels of analysis (Raudenbush and Bryk 2002).

8 Kenny (1979) developed the notion of empirical underidentification in situations in which researchers, despite assuring the identification of the model, cannot achieve a clear-cut solution for the model parameters in the analyzed **S** matrix of covariance.

9 Comments on nonlinear relationships and nonlinear interaction between variables or issues concerning methods allowing to specify and to test interaction models and nonlinear effects in reference to SEM models can be found in papers by Schumaker and Marcoulides (1998), Yang-Wallentin et al. (2004), Moulder and Algina (2002), Lee et al. (2004), Cortina et al. (2001) and Marsh et al. (2004a, b). Particular attention should be paid to the work of McDonald (1967, 1983), Mooijaart and Bentler (1986), who developed the statistical basics of models consisting of nonlinear relationships between variables and nonlinear interactions. In some part these works were connected with the work of Kenny and Judd (1984). Since then there have been many developments allowing to test mediation and moderation (interaction) in SEM which even led to a new software package based on R for nonlinear SEM.

10 For those interested in gaining more knowledge on the consequences of randomization and passive observation of causal inferences, please refer to the works of Pratt and Schalifer (1984, 1988), Sobel (1995) or Pearl (2000).

11 Unfortunately, in Hempel’s deductive-nomological formulation of the explanation the concept of causality as a form of explanation was dropped out of consideration for and replaced by the logical operation of material implication.
